# Supplementary material for: Unravelling reference bias in ancient DNA datasets
Source: Bioinformatics. 2024 Jul 3;40(7):btae436. doi: 10.1093/bioinformatics/btae436 (PMC11254355; doi:10.1093/bioinformatics/btae436)
Supplement: btae436_Supplementary_Data [file btae436_supplementary_data.zip › S1-S4_data_plots.zip!/S1-S4_data/rhino_sim_plots.zip!/rhino_sim_plots/verysensitive_bowtie_rhino_div0_1end_by_MQ.pdf]

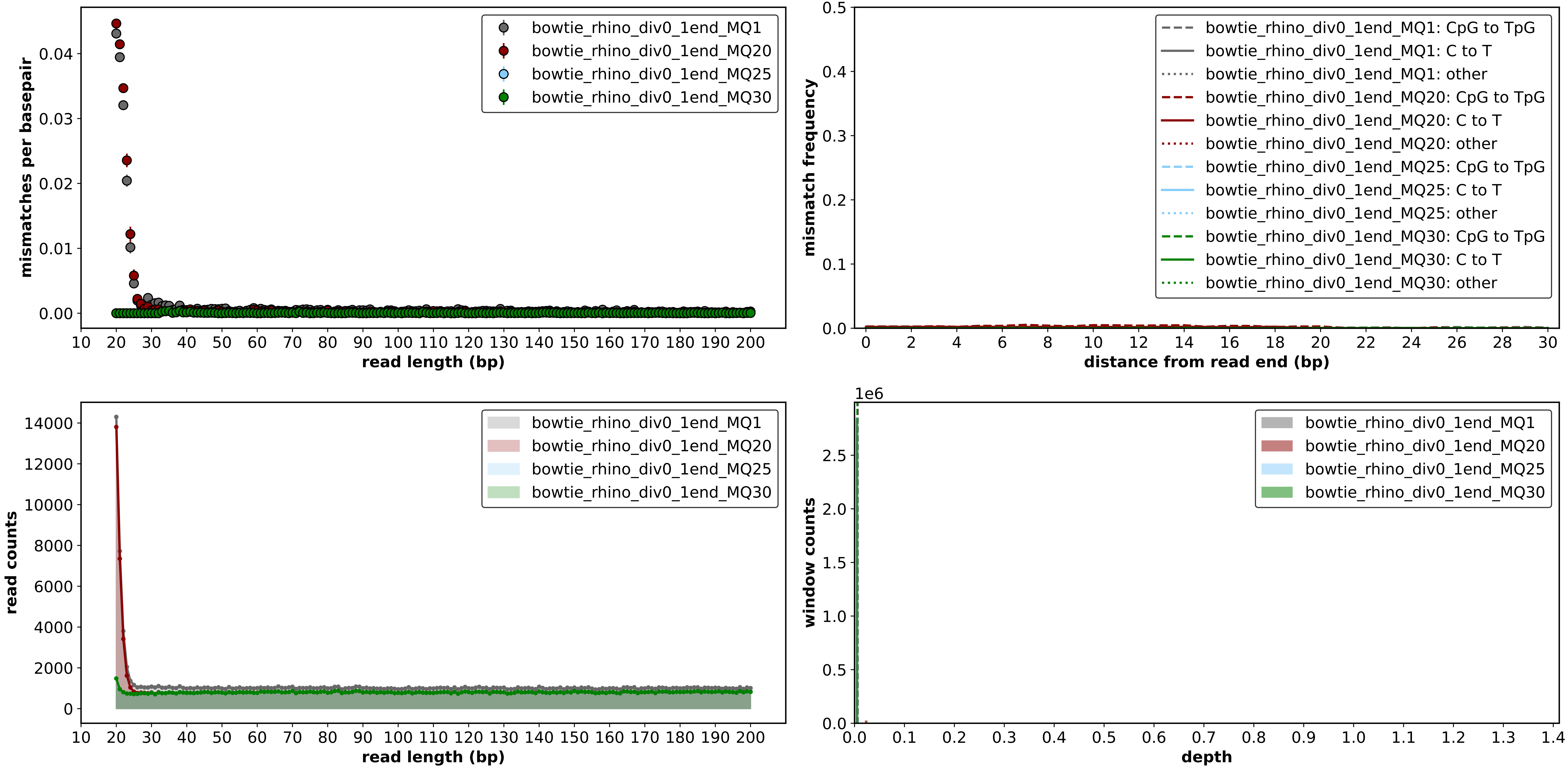

mismatches per basepair
0.04
0.03
0.02
0.01
0.00
10 20 30 40 50 60 70 80 90 100 110 120 130 140 150 160 170 180 190 200
read length (bp)
bowtie_rhino_div0_1end_MQ1
bowtie_rhino_div0_1end_MQ20
bowtie_rhino_div0_1end_MQ25
bowtie_rhino_div0_1end_MQ30
mismatch frequency
0.5
0.4
0.3
0.2
0.1
0.0
0 2 4 6 8 10 12 14 16 18 20 22 24 26 28 30
distance from read end (bp)
bowtie_rhino_div0_1end_MQ1: CpG to TpG
bowtie_rhino_div0_1end_MQ1: C to T
bowtie_rhino_div0_1end_MQ1: other
bowtie_rhino_div0_1end_MQ20: CpG to TpG
bowtie_rhino_div0_1end_MQ20: C to T
bowtie_rhino_div0_1end_MQ20: other
bowtie_rhino_div0_1end_MQ25: CpG to TpG
bowtie_rhino_div0_1end_MQ25: C to T
bowtie_rhino_div0_1end_MQ25: other
bowtie_rhino_div0_1end_MQ30: CpG to TpG
bowtie_rhino_div0_1end_MQ30: C to T
bowtie_rhino_div0_1end_MQ30: other
read counts
14000
12000
10000
8000
6000
4000
2000
0
10 20 30 40 50 60 70 80 90 100 110 120 130 140 150 160 170 180 190 200
read length (bp)
bowtie_rhino_div0_1end_MQ1
bowtie_rhino_div0_1end_MQ20
bowtie_rhino_div0_1end_MQ25
bowtie_rhino_div0_1end_MQ30
1e6
window counts
2.5
2.0
1.5
1.0
0.5
0.0
0.0 0.1 0.2 0.3 0.4 0.5 0.6 0.7 0.8 0.9 1.0 1.1 1.2 1.3 1.4
depth
bowtie_rhino_div0_1end_MQ1
bowtie_rhino_div0_1end_MQ20
bowtie_rhino_div0_1end_MQ25
bowtie_rhino_div0_1end_MQ30
